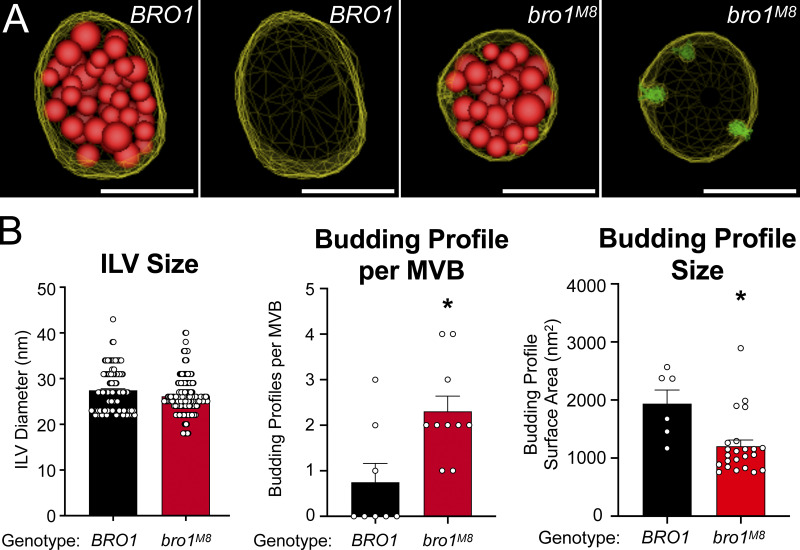# Correction: Bro1 stimulates Vps4 to promote intralumenal vesicle formation during multivesicular body biogenesis

**DOI:** 10.1083/jcb.20210207007022021c

**Published:** 2021-07-14

**Authors:** Chun-Che Tseng, Shirley Dean, Brian A. Davies, Ishara F. Azmi, Natalya Pashkova, Johanna A. Payne, Jennifer Staffenhagen, Matt West, Robert C. Piper, Greg Odorizzi, David J. Katzmann

Vol. 220, No. 8 | 10.1083/jcb.202102070 | June 23, 2021

After publication, the authors discovered an error in Fig. 7 B. The data set for a bar graph titled "Budding Profile Size" from Fig. 1 C was inadvertently duplicated in Fig. 7 B. This error was initially missed because the data point patterns of "Budding Profile Size" are similar between Figs. 1 C and 7 B. The corrected Fig. 7 panel is shown below. This change does not impact the interpretation of the figure or the conclusions of the article. The error only appears in PDF versions downloaded before July 6, 2021.

**Figure:**